# Recent Progress on Nanomaterial-Based Membranes for Water Treatment

**DOI:** 10.3390/membranes11120995

**Published:** 2021-12-20

**Authors:** Majeda Khraisheh, Salma Elhenawy, Fares AlMomani, Mohammad Al-Ghouti, Mohammad K. Hassan, Bassim H. Hameed

**Affiliations:** 1 Department of Chemical Engineering, College of Engineering, Qatar University, Doha 2713, Qatar; se1105821@student.qu.edu.qa (S.E.); falmomani@qu.edu.qa (F.A.); b.hammadi@qu.edu.qa (B.H.H.); 2 Environmental Sciences Program, Department of Biological and Environmental Sciences, College of Arts and Sciences, Qatar University, Doha 2713, Qatar; mohammad.alghouti@qu.edu.qa; 3 Center of Advanced Materials, Qatar University, Doha 2713, Qatar; mohamed.hassan@qu.edu.qa

**Keywords:** nanomaterials, membrane separation, water and wastewater treatment, membrane enhancements, nano sheets, nano composites

## Abstract

Nanomaterials have emerged as the new future generation materials for high-performance water treatment membranes with potential for solving the worldwide water pollution issue. The incorporation of nanomaterials in membranes increases water permeability, mechanical strength, separation efficiency, and reduces fouling of the membrane. Thus, the nanomaterials pave a new pathway for ultra-fast and extremely selective water purification membranes. Membrane enhancements after the inclusion of many nanomaterials, including nanoparticles (NPs), two-dimensional (2-D) layer materials, nanofibers, nanosheets, and other nanocomposite structural materials, are discussed in this review. Furthermore, the applications of these membranes with nanomaterials in water treatment applications, that are vast in number, are highlighted. The goal is to demonstrate the significance of nanomaterials in the membrane industry for water treatment applications. It was found that nanomaterials and nanotechnology offer great potential for the advancement of sustainable water and wastewater treatment.

## 1. Introduction

Water scarcity is presently a major area of concern for the entire world [1,2,3]. Rapid industrial development and global population growth are increasing the demand for several water resources. Based on UN Water ORG, 1.8 billion people are estimated to be living in areas with absolute water scarcity, and two-thirds of the global population will live under water stress conditions by the year 2025 [4]. Thus, highly efficient water treatment technologies with great sustainability are required to tackle the worldwide water scarcity issue. Membrane technology stands out to be the best technology for water treatment compared to other technologies such as distillation [5,6,7], electrolysis [8,9,10], adsorption [11,12,13], and photodegradation [14,15,16]. The reason for this is that membrane technology requires less energy to operate, attains a high separation efficiency, and is capable of operating in a continuous mode. Several studies have been conducted to increase the overall performance of membranes. Nanotechnology plays a vital role in accelerating the performance of membranes. The use of nanomaterials increases water permeability, mechanical strength, and reduces the fouling phenomenon of the membrane [17].

**Figure 1:**
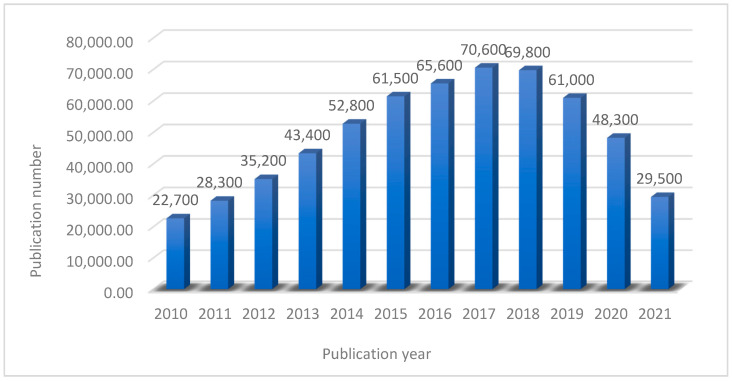
A short post-2010 timeline showing the number of water treatment nanomaterial-based membrane related academic publications.

Nanotechnology is thought to be the cure-all for the majority of problems involved with water contamination remediation. In the past few decades, the urgent need for novel membranes, made up of nanomaterials with well-defined nanostructures, has transformed traditional membrane concepts, resulting in groundbreaking water treatment methods that exceed state-of-the-art performance. The nanomaterials used in the membranes include nanoparticles (NPs), two-dimensional (2-D) layer materials, nanofibers, and other nanocomposite structural materials. Furthermore, a huge array of water treatment equipment, including those incorporating nanotechnology, are presently available in the market, with several more on the way. Figure 1 shows the number of publications about the use of nanomaterial-based membranes for water treatment.

It is clearly seen from Figure 1 that the number of publications about the use of nan-materials-based membranes in the water treatment field has a general increasing trend. This proves the importance of the nanomaterials in the membrane field for water treatment.

Several types of research and studies have been conducted to examine the use of nanomaterials in the advancement of membrane performance. This review focuses on membrane modifications following the implementation of new nanomaterials, including nanoparticles (NPs), two-dimensional (2-D) layer materials, nanofibers, nanosheets, and other nanocomposite structural materials. In addition, the applicability of these membranes containing nanomaterials in various water treatment applications are highlighted. The purpose is to prove the significance of nanomaterials in the membrane industry for water treatment applications.

## 2. Traditional Membrane Materials

Membrane technology has advanced at a breakneck pace over the last few decades [18,19]. The membranes used in industrial and laboratory separation processes are mainly made up of polymeric [20,21,22,23] and inorganic materials [24]. Generally speaking, ceramic membranes are artificial membranes synthesized by the deposition of metal hydroxides colloidal suspensions on porous supports [25]. Ceramic membranes are greatly employed in separation processes that involve strong media such as acids and strong solvents or extreme conditions such as high temperature and pressure. As a result of the accelerated chemical inertness and mechanical thermal resistance of ceramic membranes, the flux through the membrane can be recovered easily after fouling [26]. Ceramics, however, are excessively brittle, and their high production costs severely limit their application in large-scale industries [27].

Polymeric membranes, on the other hand, are the leaders in the membrane separation processes in industries due to their high performance and viable cost [28]. Porous polymer membranes are traditionally synthesized using mechanical stretching and/or a phase-inversion technique. The advantages of polymeric membranes rely upon their superb separation performance, high permeation rate, and perm-selectivity. The disadvantages include low tolerance to high-temperature levels, corrosive environments, and organic solvents [29].

Thin-film composite membranes, a significant breakthrough in the field of membranes, presently have a more flexible structure that combines a number of higher selective layers and porous support layers for a more complex separation environment [30]. Under pressure, nevertheless, the multilayer structure has significant compaction repercussions [31]. When the loading pressure is increased, the polymers rearrange into a smaller structure, resulting in a decrease in porosity and, as a result, a loss in separation efficiency. Compaction often increases with increasing pressure [32,33].

## 3. Nanomaterial-Based Membranes

**Figure 2:**
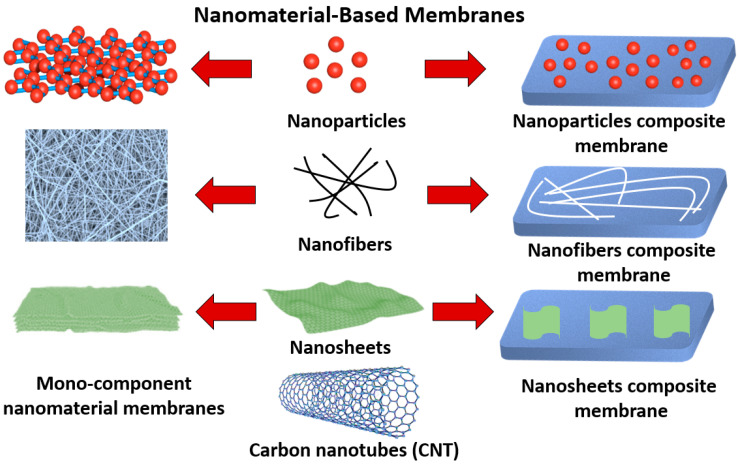
The most commonly used nanomaterial-based membrane structures.

Despite the domination of the water purification market by conventional membranes, it is difficult to choose the most preferable membrane depending on usage, because each membrane type is bounded by a tradeoff, such as selectivity, permeability, flux, stability, or high manufacturing cost [1]. In addition, fouling is a serious issue that constrains the application of ceramic and polymeric membranes. Several pathways have been taken to chemically modify the membrane surface for better performance and less fouling. Among the wide variety of the proposed technologies and pathways, nanotechnology stands out to be the most promising membrane technology. There are several nanomaterials used in membranes. Figure 2 below shows the most widely used membrane-based nanomaterials structures.

### 3.1. Nanoparticle Composed Membranes

#### 3.1.1. Freestanding Nanoparticle Membrane

Nanoparticle membranes are synthesized by assembling the nanoparticles into free-standing ultrathin membranes. Nanoparticle membranes are generated utilizing filtering, a drying-mediated self-assembly method, and blown-film extrusion, as well as nano film segregation over a two-dimensional interface created within a hole. At present, mono-component nanomaterial membranes that are entirely made up of nanoparticles such as close-packed gold nanoparticle mono-layers, are not much available. Freestanding ultrathin nano-membranes (FUN-membranes) are two-dimensional membrane materials with a nanoscale thickness of <100 nm and with very little, or almost no, substrate support. In the past few years, there has been a surge of interest in rationally designing such membranes for a wide range of applications, from electronic devices to water remediation systems [34]. However, there are few studies available in the market about the use of freestanding ultrathin nano-membranes (FUN-membranes). Zhang, et al. [35] prepared freestanding cross-linked polystyrene nanoparticle membranes that have a thickness of 80 nm and very precise pores. The membrane was synthesized by the authors via polystyrene nanoparticle filtration over a microfiltration membrane via a sacrificial layer of metal hydroxide nano strands. The synthesized membranes exhibited very interesting properties along with a quick separation of gold nanoparticles and small proteins [35]. Membrane filtration has been proposed as a viable option to remediate the environment by incorporating it into a modern oxidation processes to reduce energy and cost consumption. Ye, et al. [36] successfully synthesized a freestanding 2-D confinement graphene oxide (GO) composite membrane. The fabricated membrane had excellent capabilities of pollutant catalytic degradation. Hence, these features demonstrat great potential for the fabricated 2-D confinement catalytic membranes with enriched oxygen vacancies in wastewater purification [36].

#### 3.1.2. Nanoparticles as Filler for Composite Membrane

Filling membrane composites with nanoparticles involves the addition of nanoparticles to the ceramic or polymeric membrane during the synthesis process. The concept behind the addition of inorganic or organic materials into a polymer matrix is commonly used in the fabrication of mixed matrix membranes. In recent years, the incorporation of nanoparticles into membranes has emerged as a new focus. However, the use of these small nanosize particles in the membranes is followed by some advantages and disadvantages. The advantages include a better interaction between the two phases in the membrane which leads to higher selectivity, permeability, mechanical stability, hydrophilicity, and less fouling. In addition, some nanoparticles provide the membranes with antibacterial and catalytic properties. On the other hand, the disadvantages rely on the fact that some properties of the polymeric membranes are rendered in the presence of the nanoparticles. The most commonly used nanoparticles in the polymeric and ceramic membranes include the following: alumina, TiO_2_, silica, zinc oxide, zeolite, and attapulgite (APT) into polymeric membranes, which have shown to enhance the membrane water permeability, surface hydrophilicity, resistance to fouling, and functionalization. Table 1 below shows the membrane enhancements after the addition of some widely used nanoparticles in membranes.

Several studies have analyzed membrane efficiency enhancement after the incorporation of the most commonly used nanoparticles in several industries. Ghaemi [37] studied the improvements that occurred in the removal efficiency of copper in PES membranes after the incorporation of alumina (Al_2_O_3_) nanoparticles. The authors prepared mixed matrix membranes by a phase inversion method while using PES and various amounts of alumina nanoparticles. The results of the authors’ study show that the water permeation of the mixed matrix membranes was elevated after the addition of the alumina nanoparticles compared with the pristine PES. The increased water permeability was responsible for the increase in the porosity and hydrophilicity of the mixed matrix membrane after the incorporation of alumina nanoparticles into the matrix of the membrane. In addition, the copper ion removal efficiency of the alumina mixed membranes was also enhanced [37]. Hosseini, et al. [38] studied the enhancement effects of TiO_2_ nanoparticles on the physicochemical properties of a mixed matrix membrane. The authors used a solution casting technique to prepare polyvinylchloride-co-TiO_2_ nanoparticle mixed matrix heterogeneous cation exchange membranes. The results of the study show that the membrane ion exchange capacity, flux, mechanical strength, and selectivity were all improved after the addition of TiO_2_ into the matrix of the membrane [38]. Furthermore, in a study that included TiO_2_ nanoparticles, Mobarakabad, et al. [39] revealed the effect of titanium dioxide (TiO_2_) nanoparticles as an inorganic filler additive on the membrane physico-chemical properties. The authors used a phase inversion method to prepare asymmetric poly (1,4-phenylene ether–ether-sulfone) (PPEES)-blend-polyethylene glycol nanocomposite nanofiltration membranes with titanium dioxide (TiO_2_) nanoparticles as the inorganic filler additive and N-methyl pyrrolidone as the solvent. The results of this study revealed that there was an enhancement in the membrane water flux (WF), tensile strength, hydrophilicity, and salt rejection after the addition of TiO_2_ nanoparticles as the inorganic filler in the membrane [39]. Ayyaru, et al. [40] studied the effect of different GO-ZnO loadings on a polyvinylidene fluoride (PVDF) membrane. The addition of GO-ZnO nanocomposite significantly improved membrane porosity, wettability, water flux, and anti-fouling properties. This proves that the overall properties of the GO-ZnO/PVDF are improved compared to the bare PVDF membrane after the addition of the nanocomposite GO-ZnO [40]. Borjigin, et al. [41] studied the effect of incorporating Beta (β) zeolite in a polyamide (PA) thin-film nanocomposite (TFN) membrane. The authors found that after the modification of the TFN membrane by the Beta (β) zeolite the water flux, and the separation of the membrane significantly increased [41]. Attapulgite (APT) is a very promising highly hydrophilic mineral additive in nature that is used to modify ultrafiltration (UF) membranes. Zhang, et al. [42] used attapulgite (APT) as an additive for a polyvinylidene fluoride (PVDF) matrix to prepare a hybrid membrane using the phase inversion method. The results of the study show that the APT particle blended membranes had greater hydrophilicity, better thermal stability, higher water permeability, smaller pore size when, and enhanced antifouling performance compared with the pure PVDF sample [42]. Table 2, Table 3 and Table 4 show the enhancements in membranes after the incorporation of TiO_2_, SiO_2_, and other nanoparticles in the membranes.

#### 3.1.3. Applications of Nanoparticle Membranes in Water Treatment

The pollution of water by contaminants is a worldwide issue that must be addressed efficiently to overcome the drastic consequences of water contamination. Nanotechnology offers a wide range of applications in the field of water and wastewater treatment (Table 5).

**Figure 3:**
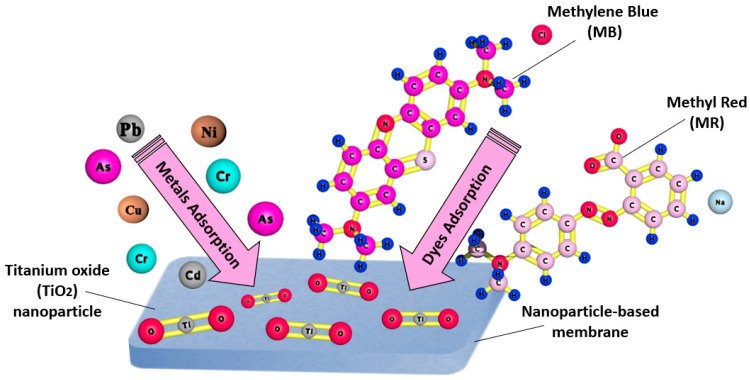
Schematic representation of heavy metals and dye adsorption by a nanoparticle-based membrane for water treatment.

**Figure 4:**
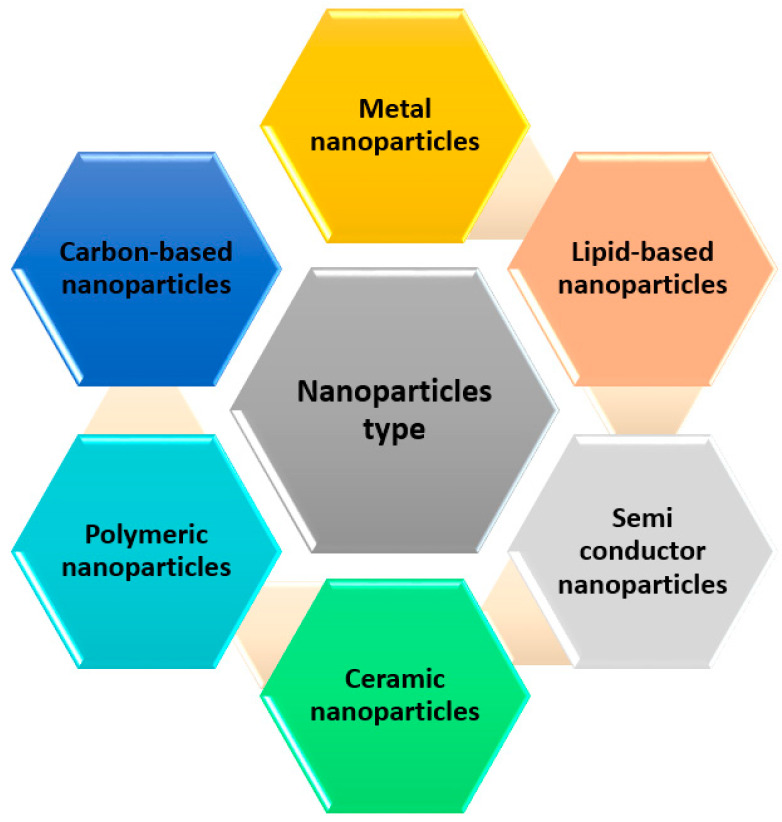
Most commonly used types of nanoparticles in water treatment.

Figure 3 represents the adsorption process of a nanoparticle-based membrane in the removal of several heavy metals and dyes for water treatment. Several studies have reported the use of nanoparticles in membranes to enhance the removal efficiency of pollutants from water. Figure 4 below shows the most commonly used nanoparticles for water treatment.

Zhao, et al. [89] added a series of defective ZIF-8 (dZIF-8) nanoparticles into polyamide-based thin-film nanocomposite (TFN) membranes for the desalination of seawater and brackish water. The authors studied the incorporation of dZIF-8 with several loadings on membrane separation performance and properties. The authors found that the separation performance and the properties of the membrane were greatly enhanced as the loadings of the nanoparticles increased [89]. The main target of Bose, et al. [90] was to fabricate a polymeric nanocomposite membrane with a low-budget nanoparticle for an effective oil-water separation process. The authors used a cellulose acetate (CA) polymer to fabricate the membrane and silicon carbide (SiC) nanoparticles to modify the membrane. The effect of silicon carbide (SiC) nanoparticle addition on membrane properties was analyzed by the authors. The results of the authors’ study show that the addition of silicon carbide (SiC) nanoparticles increased membrane hydrophilicity, pore size, water flux, porosity, and water content. An 89% increment of the pure water flux occurred after using the modified membrane. Furthermore, the antifouling properties of the membrane was enhanced as well. Besides, reasonable improvement in the antifouling attributes of the membranes were also observed. Thus, the modified membrane by the SiC nanoparticles is more efficient for the oil-water separation process than the unmodified membrane [90].

Wen, et al. [91] successfully modified a graphene oxide membrane (GOM) by super-hydrophobic modification using fluorinated silica nanoparticles layers on the membrane surface to improve the surface adhesion and decrease the surface energy. The authors used light/heavy water as a model and an air gap membrane distillation (AGMD) apparatus to evaluate the separation performance of the isotopic hydrogen of this composite membrane. The results of the authors’ study demonstrate that the selectivity of the membrane was enhanced by the addition of the fluorinated silica nanoparticles [91]. Membrane fouling is considered the main limitation to the performance of membranes. Kazemi, et al. [92] aimed to enhance the antifouling properties of a PVC membrane by incorporating GO and GO-ZnO nanoparticles into the PVC membrane in oily wastewater treatment. The results of the study revealed that increasing the nanoparticle content of the membranes improved the membrane’s hydrophilicity. In addition, the water flux, and mechanical strength of the membrane were also increased. Furthermore, the PVC/GO-ZnO membranes showed a greater turbidity removal efficiency and less flux reduction compared to the PVC and PVC/GO membranes [92]. There is a wide range of emerging technologies for the treatment of oily wastewater using ultrafiltration membranes that use hydrophilic nanoparticles for improving membrane efficiency. De Guzman, et al. [93] fabricated cellulose acetate (CA) mixed-matrix membranes with zwitterionic nanoparticles (polydopamine-sulfobetaine methacrylate P(DA-SBMA)) via a wet-phase inversion method for treating oily wastewater. The authors’ found that the addition of the nanoparticles improved membrane porosity, hydrophilicity, water flux, flux recovery, and reversible fouling. The authors used several oil-in-water emulsions in their study, including containing diesel oil, toluene, hexane, dodecane, and food-grade oil. The study revealed that oil-water separation efficiencies from 95% up to 99% were achieved. Thus, nanoparticles were successful in improving the performance of membranes for oily wastewater treatment [93].

The design and fabrication of polymeric membranes with high rejection and outstanding permeability remains a major issue. Zhang, et al. [94] synthesized and used metal-organic framework nanoparticles that are soluble in water to modify a polyether-sulfone membrane forming a uniform porous membrane. The results of the authors’ study showed that the permeability of the modified membrane was enhanced considerably. In addition, the water flux was also significantly enhanced. Furthermore, the modified membrane had a high rejection of approximately 100% for bovine serum albumin. Thus, the modification of the membrane with the nanoparticles significantly improved its separation performance [94]. Zhao, et al. [95] incorporated UiO-66-NH_2_ nanoparticles into polyamide-based thin-film nanocomposite (TFN) RO membranes. The outstanding properties of the UiO-66-NH_2_ nanoparticles enhanced the membrane surface hydrophilicity and decreased the preferential pathways and degree of cross-linking for the water molecules across the selective layers. In addition, the TFN membranes showed a higher salt rejection and water flux compared to the benchmark membranes [95]. Kotp [96] reported a new method containing high flux thin-film nanocomposite (TFN) nanofiltration (NF) membranes. The authors synthesized the membranes by incorporating camphor-Al_2_O_3_NPs (CA.TFN) and commercial-Al_2_O_3_ (CO.TFN) into polyamide layers using an interfacial polymerization method. The results of the study revealed that the addition of the camphor-Al_2_O_3_ NPs into the TFC membrane improved membrane water flux, salt rejection, and hydrophilicity [96]. In a further study, Matindi, et al. [97] fabricated polyethersulfone (PES)/sulfonated polysulfone (SPSf)/TiO_2_ mixed matrix membranes (MMMs) for the oil/water emulsion separation process. The authors analyzed the membrane performance by various loadings of TiO_2_ nanoparticles (NPs) and polymer concentrations. The results of the authors’ study exhibited that adding small concentrations of TiO_2_ NPs into the membrane led to an outstanding improvement in the separation performance of membranes applied in oil/water emulsion filtration [97]. Barati, et al. [98] used in situ grown iron oxide nanoparticles (NPs) study to impregnate commercial ceramic membranes via a facile technique to treat produced water. The results of the authors’ study revealed that membrane hydrophilicity, organic rejection, and antifouling behavior were improved significantly after the addition of the iron oxide nanoparticles (NPs) [98].

The use of nanocomposite adsorptive membranes that incorporate nanosorbents is a very promising option for water treatment from heavy metals; however, the aggregation of nano-sorbents in the membrane matrix has hampered their practical uses. He, et al. [99] prepared an adsorptive membrane made up of homogenous in-situ generated ferrihydrite nanoparticles (NPs)/polyethersulfone (PES), and strived to remove lead from water containing heavy metals. The synthesized membrane had high surface hydrophilicity and water flux. In addition, it also showed high adsorption capacity and selectivity of Pb^2+^, and outstanding reusability without significant loss of Pb^2+^ adsorption. Consequently, the reported membrane in this study with the ferrihydrite nanoparticles (NPs) is a very promising present material for the removal of heavy metals from water [99].

There have been huge advancements using inorganic membranes in the treatment of marginal water containing hydrocarbon contaminants. Liu, et al. [100] incorporated silica nanoparticles into an alumina matrix to achieve hydrophilic modification of alumina microfiltration membranes. The alumina membrane incorporating silica nanoparticles was intended to separate cyclohexane from water. The study demonstrated that the added silica nanoparticles significantly increased membrane hydrophilicity, water flux, and oil rejection. This study proved that the addition of nanoparticles in the membrane enhances the overall performance of the membrane with respect to oil-water separation processes [100]. The separation of oil-water emulsions can be successfully achieved by using porous ceramic membranes with great mechanical strength. However, the preparation of ceramic membranes that have small pore sizes and remarkable antifouling properties is quite hard to attain. Zhang, et al. [101] modified β-SiAlON ceramic membranes with SiO_2_ nanoparticles for the removal of oil droplets from an oil-water emulsion. The modified membranes had a very small pore size, and water fluxes that were outstanding for the oil-water separation process. Furthermore, the membrane displayed a high oil rejection rate and remarkable antifouling ability. Thus the synthesized membrane in this study with the nanoparticle can be considered as a promising material for oil-contaminated wastewater treatment [101]. Rowley and Abu-Zahra [102] used Fe_3_O_4_ nanoparticles (NPs) to modify polyethersulfone (PES) nanocomposite membranes for the removal of arsenic from water. The fabricated PES membranes with A-Fe_3_O_4_ NPs showed a high adsorption capacity of arsenic from water using only small concentrations of the A-Fe_3_O_4_ NPs. The results prove that the synthesized membrane in this study with the incorporated A-Fe_3_O_4_ NPs is a very efficient candidate for the treatment of water from arsenic [102]. Table 6 below shows the applications of several nanoparticles for the removal of contaminants from water.

### 3.2. Nanofiber-Composed Membrane

#### 3.2.1. Freestanding Nanofiber Membrane

Nanofibers are traditionally stated as fibers with diameters less than 100 nm. Nanofibers are known for having a high weight ratio, and highly porous structure with remarkable pore interconnectivity [130]. The ratio of the nanofibers is very high, which makes it easier for them to interlock and form a freestanding porous membrane. The distinguishable properties of the nanofibers allow them to be used in various applications in several industries.

Cellulose fiber-modified membranes have been used for a long time ago in various water treatment applications. A new cellulose form was discovered in the last century that allows the design of new liquid separation membranes. Cellulose has outstanding film-forming and mechanical properties that enable it to be used in several industries. In addition, the cellulose surface is easy to modify and very safe to use, which eases the process of surface modification.

In many separation processes, there is an increasing demand for solvent-resistant and highly efficient nanoporous membranes. It is common for membranes to have a low permeation flux as a result of a low resistance to solvent and a thick membrane layer. The synthesis of ultrathin nanometer pore size membranes for rapid organic filtering is now the most difficult issue. Zhang, et al. [131] prepared ultrafine cellulose nanofibers via a facile method for the fabrication of ultrathin nano-porous membranes. The synthesized nanofibers had a diameter of 7.5 ± 2.5 nm, and the cellulose nanoporous membranes had an adjustable thickness down to 23 nm. In addition, the cellulose nano-porous membranes had very narrow pore sizes that ranged from 2.5 to 12 nm. The resultant nanocellulose membrane had rapid permeation of water and several organic compounds in a pressure-driven filtration process. In addition, the prepared cellulose nanofibers in this study were easy to use in the production of novel syringe filters with less than 10 nm pore size, which has a wide range of applications in the rapid separation and purification process [131].

The fabrication of other biopolymer-based nanofiber ultrathin membranes was the main aim of several studies. Ling, et al. [132] synthesized a new ultrathin filtration membrane made of silk nanofibrils (SNFs), which were exfoliated from natural *Bombyx mori* silk fibers, for the separation of various dyes, proteins, and colloids of nanoparticles. The synthesized membranes had a thickness down to 40 nm and pore sizes ranging from 8 to 12 nm. The SNF-based ultrathin membrane synthesized in this study showed a water flux of 13,000 L·h^−1^·m^−2^·bar^−1^, which is greater than 1000 times of the most commercial ultrathin filtration membranes at present. In addition, the SNF-based ultrathin membranes exhibited very high efficiency for dyes, colloids of nanoparticles, and proteins, with a minimum of 64% rejection for Rhodamine B. Thus, the reported SNF-based ultrathin membrane in this study is a promising material for a broad range of applications in water and wastewater treatment.

Various techniques can be used in the preparation of nanofibers, including melt-blowing, flash-spinning, splitting of bicomponent fibers, physical drawing, phase separation [5], self-assembling [6], centrifugal spinning, solvent dispersion [7,8], hydrothermal [9], and electrospinning. Electrospinning is the best method of nanofiber preparation among all the mentioned methods. Electrospinning surpasses the mentioned methods by its high versatility in the preparation of nanofibers using a wide range of materials, and the capability of controlling the nanofiber diameter, morphology, and structure. In addition, it is easy to modify by the addition of several nanomaterials or soluble substances to the electrospinning solution. However, the application of electrospinning is hindered by its high cost, since it needs massive-scale solvent recovery from a dilute air stream, making the process uneconomical. Several studies reported the use of electrospinning for the preparation of nanofiber membranes for water treatment applications. Du, et al. [133] prepared via one-step electrostatic spinning of a polyvinylpyrrolidone (PVP), polyvinylidene fluoride (PVDF), and an inorganic titanium dioxide (TiO_2_) nanoparticles blend nanofiber membrane. The presence of PVDF increases the strength and chemical resistance of the membrane. In addition, the PVP enhances the hydrophilicity and the mechanical strength of the membrane. The synthesized membrane in this study showed a high separation efficiency (98.4%) for various emulsions, great antifouling properties with a remarkable flux recovery rate (FRR 95.68%), and a low total fouling ratio (15.18%) after many cycles. Thus, the electrospinning method is a remarkable method for the preparation of nanofiber membranes to be used in water treatment processes [133].

#### 3.2.2. Nanofibers as Filler for Composite Membranes

In the water purification field, thin-film composite membranes (TFC) have received a lot of researchers’ attention. Using a one-step procedure, a unique TFC membrane was created based on a layer of polyvinylidene fluoride (PVDF) that formed tree-like electrospun nanofiber membranes (TENMs) [134]. The TENMs were characterized by a high-proportion of interconnect pores, high surface porosity, a pore size less than 200 nm, and low tortuosity, compared to the traditional support membranes. Thus, this is very promising for the fabrication of high-performance TFC nanofiltration (*NF*)membranes. The results of the authors’ study revealed that the rejection rate was greater than 97% against the MgSO_4_ solution and 76% against NaCl solution, showing great potentials in the water purification field [134].

As a result of the remarkable sieving performance for small molecules and ions, lower energy requirements, and high permeation flux, nanofiltration plays a major role in a wide range of processes. On the other hand, current nanofiltration membranes (NFMs) face significant difficulties in improving permeability while keeping a high rejection rate for divalent (or multivalent) ions. Lv, et al. [135] used an electrospun polyacrylonitrile nanofiber membrane as a support for a fabricated thin-film composite (TFC) nanofiltration membrane. The resulting NFMs had high water flux along with an excellent rejection rate for divalent anions and cations. This study opens the door for highly efficient methods for the preparation of NFMs to be used in various separation applications [135].

#### 3.2.3. Applications of Nanofiber Membranes in Water Treatment

Catastrophic oily discharges into water are a huge concern for environmental pollution. Effective electrospun nanofiber membranes have attracted great interest due to their high surface area, high porosity, customizable wettability, and uniform pore distribution [136]. However, the most frequently used nanofiber membrane modification methods, including grafting and surface coating, are strictly limited, and thus reduce their use in several applications. Thus, Du, Wang, Liu, Wang and Yu [133] used a one-step electrostatic spinning technique to fabricate a polyvinylpyrrolidone (PVP), polyvinylidene fluoride (PVDF), and inorganic titanium dioxide (TiO_2_) nanoparticle blend nanofiber membrane. The addition of these chemicals increased the nanofiber membrane mechanical strength, hydrophilicity, and chemical resistance. In addition, the membrane appeared to be oleophobic in water and hydrophilic in air. The results of this study show that the produced membrane attained a separation efficiency of 98.4% for several emulsions, distinguishable antifouling properties with a high flux recovery rate reaching 95.68%, and a low total fouling ratio up to 15.18%, after its use in several cycles. Thus, the fabricated membrane is an excellent choice for oil/water separation applications [133]. Xu, et al. [137] synthesized a unique tubular polyvinyl chloride (PVC) hybrid nanofiber membrane using hydrophobic nanosilica (SiO_2_) as the inorganic additive and a polyester (PET) hollow braided tube as the support. The fabricated membrane appeared to be very efficient in the separation of liquid, because of its remarkable separation efficiency for various water/oil emulsions. Furthermore, the membrane showed a distinguishable superhydrophobicity and lipophilicity under oil. In addition, the membrane had a high permeation flux and a remarkable separation efficiency greater than 95% under gravity. In addition, the three-dimensional tubular nanofiber membrane showed excellent porosity, mechanical properties, thermal stability, and hydrophobic stability. All of the above-mentioned properties of the synthesized three-dimensional tubular nanofiber membrane allow it to be effectively used in oily wastewater remediation processes [137]. As a continuation to oily wastewater separation methods, Su, et al. [138] fabricated a poly (vinylidene fluoride-co-hexafluoropropylene) (PVDF-HFP) nanofiber (PNF) membrane via a solution blow spinning technology. The fabricated membrane had high membrane roughness, super hydrophobicity and super lipophilicity. In addition, the membrane was capable of separating various oil/water mixtures including toluene, n-hexane, dichloromethane, and kerosene by gravity with a high (toluene/water) separation efficiency up to 99.99. Furthermore, the membrane had the capability of degrading organic pollutants in oily wastewater. Thus, the synthesized membrane is fully capable of being used as an efficient candidate for oil/water treatment processes [138]. Zhang, et al. [139] fabricated a PVDF/graphene (GE) composite membrane (TPGCM) covered with micro/nanospheres and tubular nanofibers. The membrane showed high superoleophilicity in air, remarkable separation efficiency, and outstanding recyclability. Thus, it can be efficiently used in oily water treatment industries [139]. Obaid, et al. [140] fabricated an electrospun nanofiber membrane (ENM) that is super-hydrophilic and, underwater, super-oleophobic. The nanocoated-ENMs synthesized in this study showed excellent oil/water emulsion separation performance. In addition, the nanocoated-ENMs had high flux and separation efficiency for a surfactant-stabilized oil-in-water emulsion up to 97.5%. Furthermore, the nanocoated-ENMs showed outstanding chemical stability, reusability, and durability in harsh environments. Thus, the synthesized nanocoated-ENMs are promising candidates for oil/water emulsion separation [140].

Superwetting interfacial porous membranes with several wettabilities can be widely used in wastewater treatment. Several factors control membrane wettability including pH, temperature, and pressure. Yin, et al. [141] used a calcining-spraying method to prepare a novel electrospun SiNPs/ZnNPs-SiO_2_/TiO_2_ (SZST) nanofiber membrane. The resultant membrane had the capability of changing its wettability in several environments. The membrane showed a high separation efficiency up to 99% for an oil/water emulsion. Furthermore, the membrane had excellent chemical stability and corrosion resistance. Consequently, the synthesized SZST nanofiber membrane in this study can be efficiently used in various water treatment processes [141]. Venkatesh, et al. [142] fabricated a DTPA/MWCNT/TiO_2_-polyvinylidene difluoride (PVDF) nanofiber membrane for oil-in-water emulsion separation. This showed a good underwater oleophobicity and hydrophilicity, and high separation efficiency for oil-water emulsions. Thus the synthesized nanofiber membrane in this study has great potential in oil-water treatment processes [142]. Wang, et al. [143] synthesized a deacetylated cellulose acetate (d-CA) nanofiber membrane for oil/water separation. The fabricated membrane was super-hydrophilic in oil and oleophobic in water and showed high separation efficiency of 99.97% and separation flux of 38,000 L/m^2^·h. The d-CA nanofiber membranes showed outstanding self-cleaning and antipollution abilities. Thus, the synthesized nanofiber membrane in this study can be efficiently used in the separation of oil from water [143]. The usage of electrostatic spinning to fabricate nanofiber membranes (NFMs) has gained great interest in the treatment of wastewater due to these membranes having a large specific surface area and high porosity. However, the large-scale application of nanofiber membranes (NFMs) is limited by fouling and their incapability of removing very small molecular weight dyes. Thus, Li, et al. [144] fabricated polyacrylonitrile (PAN)-ZnO NFM for efficient dye removal. The results of the study demonstrated that the (PAN)-ZnO NFM exhibited a very high removal rate of more than 95% for sunset yellow (YS), methylene blue (MB), Congo red (CR), rhodamine B (RhB), and methyl orange (MO) with an outstanding water flux (1016 L·m^−2^·h^−1^·Bar^−1^. In addition, the PAN-ZnO NFM had remarkable mechanical properties and antifouling abilities. As a result of the excellent abilities that the PAN-ZnO NFM holds, it is fully capable to be used in dye removal from wastewater [144]. Ozbey-Unal, et al. [145] synthesized hydrophobic nanofiber membranes to remove salt and boron from geothermal water using air gap membrane distillation (AGMD). The results of the study showed that the permeate flux and the mechanical strength of the membrane were improved. Hence, the membrane was capable of removing salt and boron from geothermal water [145]. Wang, et al. [146] synthesized a multifunctional polyvinylidene fluoride-co-hexafluoropropyle (PVDF-HFP)/catechol-polyethyleneimine (CA-PEI)/Ag/3-glycidyloxy propyltrimethoxysilane (KH560) tubular nanofiber membrane (TNM) for oil/water separation and dye degradation. The results of the study show that the fabricated membrane had a high separation rate and separation efficiency along with a catalytic ability for the degradation of several dyes. Thus, the membrane is efficient for oil/water separation and water treatment processes [146].

The development of a cost-effective and fast-paced oil/water separation process has become necessary due to an increase in oil spills and significant organic contamination of the marine environment. Moatmed, et al. [147] introduced flexible and freestanding hybrid polystyrene nanofibers as a hybrid membrane for ultrafast oil/water separation. The authors used several loadings of Fe_3_O_4_ nanoparticles and added them to the polystyrene nanofibers to synthesize a superhydrophobic/super-oleophilic membrane. The results of the study show that the addition of (Fe_3_O_4_) nanoparticles to the membrane improved the separation efficiency and superhydrophobic properties of light and heavy oils. The synthesized membrane had a very high flux (5000 L·m^−2^·h^−1^), along with a separation efficiency of up to 99.8% for hexane. Thus, the nanofiber membrane synthesized in the current study can be efficiently used in oil/water separation industries [147]. Choi, et al. [148] aimed to adsorb heavy metal ions from water by synthesizing a thiol-functionalized cellulose nanofiber membrane. The membrane showed a high adsorption rate for Cd(II), Cu(II), and Pb(II) ions. Thus, the synthesized membrane can be used in the remediation of water from heavy metal ions [148]. Zhang, et al. [149] fabricated an alkali lignin/poly (vinyl alcohol) (lignin/PVA) composite nanofiber membrane for the adsorption of Safranine T (ST). The nanofiber membrane showed excellent adsorption ability for Safranine T (ST) from water. Hence, the synthesized alkali lignin/poly (vinyl alcohol) (lignin/PVA) composite nanofiber membrane can be used as an efficient adsorbent for dyes from wastewater [149].

The fabrication of highly porous super-hydrophobic and super-oleophilic materials is very important in the efficient removal of oils and dyes from wastewater. Gao, et al. [150] synthesized a hybrid nanofiber membrane (FHNM) containing SiO_2_/polyvinylidene fluoride (PVDF) microspheres for oil separation from water. The results of the study showed that the FHNM was fully capable of separating oil, and corrosive solutions from water. Hence, FHNM is a promising candidate for oil remediation from water [150]. Cao, et al. [151] prepared a stellate poly(vinylidene fluoride) (PVDF)/polyethersulfone (PES) microsphere-nanofiber membrane for oil/water separation. The synthesized PES/PVDF membranes showed excellent ability in the separation of oils from water. Hence, the PES/PVDF membranes mentioned in this study can be successfully used in oil/water separation processes [151]. Zhang, et al. [152] synthesized a TiO_2_ nanofiber membrane for water treatment. The TiO_2_ nanofiber membrane appeared to have a high Humic Acid removal reaching 90%. Thus, the synthesized TiO_2_ nanofiber membrane is a promising candidate for water treatment [152]. Table 7 and Table 8 below shows the application of several nanocellulose and nanofibrous membranes in the removal of contaminants from water.

### 3.3. Two-Dimensional Layer Materials Composed Membrane

In today’s society, studies in the fields of the chemical industry, energy conservation, and environmental remediation are all confronting significant hurdles in terms of the usefulness, durability, and performance of essential main materials. It is well known that complex and advanced carbon-based nanomaterials, such as graphene, will keep on evolving and accelerating over time. These carbon-based nanomaterials are predicted to play a key role in resolving several important difficulties and achieving advances in engineering and technology. These carbon nanomaterials are used in several water treatment applications such as dye removal, oil separation from water, and heavy metal ions removal.

**Figure 5:**
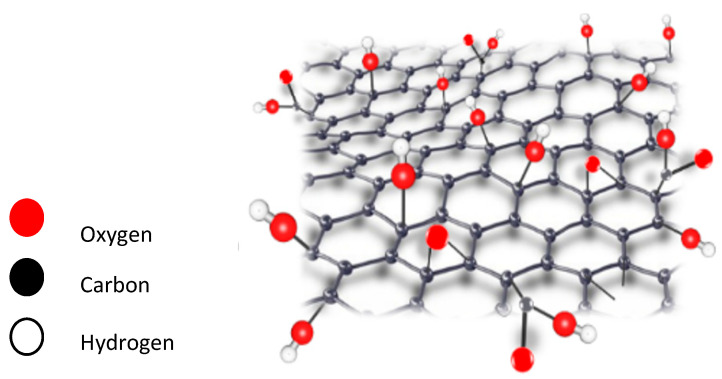
Graphene three-dimensional (3-D) structure.

In the membrane field, two-dimensional materials (2-D) have evolved rapidly in chemical engineering research and water treatment applications. It all started in the year 2010 when Geim and Novoselov were awarded the Nobel Prize in Physics for their groundbreaking experiment in the two-dimensional graphene [223]. Graphene and other two-dimensional materials are the main focus of a wide number of studies in several research fields. According to Whitby [224], graphene has a honeycomb crystal structure made up of a monolayer of carbon atoms with a one-atomic thickness of sp^2^ linked carbon that forms a two-dimensional (2-D) array of carbon atoms arranged in a hexagonal structure. The unique characteristics of two-dimensional carbon-based materials, especially graphene, makes them the ultimate choice for membrane materials. These 2-D materials have a two-dimensional structure with a mono-atomic thickness, high chemical inertness, and mechanical strength. Typically, graphene has been regarded as an ideal membrane because of its monolayer structure with mono-atomic thickness. Figure 5 below shows the three-dimensional structure of graphene.

The wide variation in 2-D materials opens the door for a lot of possibilities for the development of two-dimensional-material membranes (2DMMs). Porous graphene, zeolite nanosheets, and MOFs can all be used to produce nanosheet membranes. The strategic selection of the aperture size and porosity of in-plane nanopores can significantly improve the membrane’s selectivity and permeation simultaneously. However, nonporous nanosheets, such as graphene oxide (GO), can be drawn into laminar membranes with ordered structures. Basically, defect-free graphene is an impenetrable material for all types of molecules, even for the tiniest ones, thus it is crucial to either drill nano-size pores in graphene nanosheets or construct the nanosheet into a laminar membrane to give it the size-sieving feature. Several molecular dynamics simulations and tests have proved the feasibility of using graphene ultrathin membranes with functionalized nanopores for the separation of various ions and liquids with a remarkable selectivity and a fast separation rate. However, large-scale production of graphene nanosheets functionalized with nanopores is very difficult, since it requires very precise control for correct membrane pore size and distribution. In addition, as the applied pressure exceeds the critical pressure, the produced nanopores may undergo a loss of mechanical strength and stability, leading to catastrophic ripping of the membrane. With the great number of challenges facing the use of graphene nanosheets functionalized with nanopores, most of the studies primarily focus on finding an explanation for the transport behavior and sieving mechanism of the membrane. On the other hand, graphene derivatives, including a graphene oxide (GO) membrane and reduced graphene, do not face the same problems as graphene nanosheets with functionalized nanopores. Because of the nanochannels in their membranes, the nanopores in their nanosheets, and their functional groups, graphene derivatives have strong selective separation capability.

The presence of the oxygen-containing functional groups in graphene oxide nanosheets enables them to be formed into a laminar structure membrane using several techniques such as vacuum filtration, and dip coating. Many studies have focused on improving the structural stability of graphene oxide nanosheets, such as the addition of metal ions or molecules to the nanosheets. Furthermore, controlling the graphene-based membranes pore sizes is a necessity for the exploration of their transport characteristics and their application in several water treatment fields, such as water desalination. Based on present research studies, graphene oxide (GO) membranes hold very promising desalination capabilities owing to their easy fabrication process, low cost of production, and great performance [225]. Chen, et al. [226] synthesized ultrathin graphene membranes with very precise control of subnanometer pores via coassembling a graphene oxide nanosheet and a polymer on a porous ceramic substrate. The results of the authors’ study show that the synthesized graphene membranes have distinguishable molecular-sieving water evaporation properties that achieve a very high water evaporation flux compared to other conventional membranes. Thus, the graphene membrane fabricated in this study is a very promising material for water desalination and other separation processes [226]. The remediation of detrimental heavy metals from the marine environment has become the focus of a wide number of studies as a result of the catastrophic implications that they have on the whole environment and human body. 

Modi and Bellare [227] fabricated a unique nanohybrid that compriseszeolitic imidazolate framework-67 nanoparticles-decorated carboxylated graphene oxide nanosheets (ZIF-67/cGO) incorporated in polyethersulfone (P) hollow fiber membranes (HFMs) to improve membrane separation efficacy. The results of the authors’ study showed that the addition of ZIF-67/cGO nanohybrid in HFMs enhanced the physicochemical properties of the nanocomposite (ZcGP) HFMs, which led to a remarkably high pure water flux (346.4 ± 11.2 L/m^2^/h) and an outstanding flux recovery (95.7%). In addition, the membrane showed high adsorption capacity and removal of Cu^2+^ and Pb^2+^ heavy metal ions from contaminated water. Thus, the membrane provided in this study is an efficient material for the separation of heavy metals from water [227].

Current studies have used two-dimensional materials other than carbon-based 2-D materials, such as carbon nitride nanosheets (g-C3N4NSs), 2-D boron nitride nanosheets (BNNS), and metal-organic framework nanosheets in water treatment applications. In addition, a few studies have reported the use of mixed matrix membranes that comprise nanosheets in water remediation. Amid, et al. [228] fabricated ultrafiltration polycarbonate mixed matrix membranes (MMMs) for the separation of oil from water. Graphene oxide nanosheets and modified halloysite nanotubes were incorporated by the authors into the blank membrane. The result of the authors’ study show that after the modification of the membrane, the oil rejection rate and oil removal efficiency were enhanced. Thus, the synthesized MMMs in this study are a promising candidate for oil/water separation processes [228].

#### Application of Two-Dimensional Layer Materials Composed Membrane in Water Treatment

**Figure 6:**
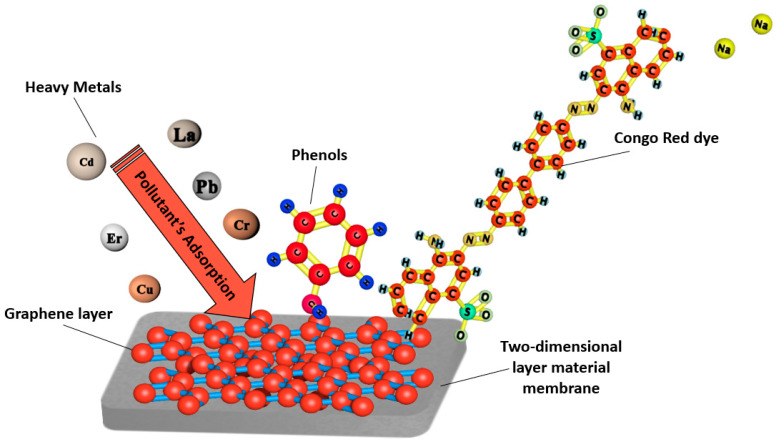
Schematic representation of heavy metals, dyes, and phenols adsorption by a graphene-based membrane for water treatment.

Currently, two-dimensional layer materials composed of membranes are becoming new-generation materials for water treatment applications with high efficiency (Figure 6). Several studies have focused their search on graphene oxide (GO) membranes for a wide variety of water treatment applications. The unique two-dimensional GO membrane interlayer nanostructure provides a base for a very precise and efficient molecular sieving for rapid water and ion transport applications. However, there are a few studies concerning the transport mechanism of water and ions through the GO membrane’s 2-D interlayer nanochannels. In addition, the GO membrane application in several water treatment processes is limited by the tradeoff between selectivity and permeability. Li, et al. [229] investigated the water and ion transport mechanisms in the two-dimensional nanochannels of the GO membrane for the development of GO membranes for a desalination process. The authors found there was an interaction between the oxygen-containing groups in the GO nanosheets and the water/ion, which proved there was successful transport. Thus, GO membranes was efficiently used in the desalination process [229]. The major industrial effluent that pollutes the environment is oily wastewater. Membrane technology is widely used in the treatment of oily wastewater to limit its catastrophic effects on the environment. Zeng, et al. [230] synthesized Hal@MXene-PDA two-dimensional (2-D) composite membranes via vacuum filtration to investigate their application in oil/water separation. The Hal@MXene-PDA composite membrane showed higher hydrophilicity compared to the bare membrane. In addition, the membrane showed high pure water flux and high oil rejection (petroleum ether and lubricating oil) up to 99.8%. Furthermore, the modified membrane(M6) also had excellent anti-fouling abilities. Thus, the membrane fabricated in this study is a promising candidate for oil-water separation [230]. Feng, et al. [231] synthesized a reduced graphene oxide (RGO)/polydopamine (PDA)/titanium carbide (MXene) composite via a dopamine modification approach. The authors suction filtrated the RGO/PDA/MXene composites on a nylon membrane to fabricate a two-dimensional-two-dimensional (2D-2D) laminated composite membrane. The modified membrane demonstrated very high removal, greater than 96% for the following dyes: Methylene Blue (MB), Methyl Red (MR), Methyl Orange (MO), Evans Blue (EB), and Congo Red (CO). In addition, the modified membrane also exhibited a very high oil/water separation greater than 97% on emulsions. Moreover, long-term cycle experiments conducted by the authors demonstrated the stability of RGO/PDA/MXene composite membranes. Thus, the RGO/PDA/MXene composite membranes synthesized in this study is a very promising applicant in the oil/water separation process and water treatment field [231]. Zhao, et al. [232] synthesized a graphitic carbon nitride nanosheet/reduced graphene oxide/cellulose acetate composite photocatalytic membrane (g-C_3_N_4_ NS/RGO/CA) for water treatment applications. Under visible light irradiation the membrane exhibited an outstanding performance in water treatment. The membrane showed high removal efficiency of Rhodamine B and excellent anti-fouling property. The membrane also had high removal efficiency for COD_Mn_, UV_254_, TOC, and bacteria from the surface of the water. Thus, the synthesized membrane in this study can be successfully used in water treatment applications [232].

Small pollutants and organic molecules cause detrimental environmental effects that destroy the environment and human health. However, their removal from the environment is very difficult due to their narrow size. Yang, et al. [233] demonstrated single-layer nanoporous graphene (NPG) membranes for the removal of organic pollutants (methanol, ethanol, urea, n-nitrosodimethylamine (NDMA), 2-propanol, pyrrole, and phenol) from water. The nano porous graphene (NPG) membranes exhibited high water permeability and selectivity against the target organic pollutants. Thus, the proposed membrane in this study is a promising candidate for the removal of organic contaminants from water [233]. Nanomaterials are mainly incorporated into the membranes to enhance the membrane permeation flux and oil/water emulsion separation performance. Zhang, et al. [234] fabricated nanocomposite membranes graphene oxide/halloysite nanotubes (GO/HNTs) that consisted of GO nanosheets and HNTs. The fabricated membrane exhibited high permeation flux and rejection rate. In addition, the GO/HNTs composite membrane was successfully used for oil-water separation experiments. Consequently, the proposed membrane in this study is a promising candidate for oil/water separation processes [234]. Table 9 below shows the application of some graphene and its derivatives in the removal of heavy metals and dyes from water.

## 4. Conclusions

The fast pace of industrial development and e global population growth is increasing the demand for several water resources. Thus, high-performance water treatment technologies are required. Membrane technology is the best technology for water treatment compared to other conventional technologies. However, application is hindered by several factors including fouling, selectivity, and permeability. Consequently, the use of materials to improve the performance of membranes is required.

Nanomaterials have emerged as the new future generation materials for high-performance membranes that are expected to solve the water crisis issue. The use of nanomaterials increases water permeability and mechanical strength, and reduces fouling of the membrane.

This review paper highlights the incorporation of several nanomaterials in membranes in various water treatment fields. It is recommended that researchers and scientists should apply more effort in the field of nanomaterials and try to reduce the overall cost of the process. Issues related to the scale up of the production of nanomaterials and their derivatives, and applications in situ, are also important aspects for future development. In addition, the toxicity of the nanomaterials themselves may need further investigation.

## Figures and Tables

**Table 1:** Three-dimensional structures and membrane enhancements for some widely used nanoparticles in membranes.

Nanoparticle	Three-Dimensional Structure (3-D)	Enhancements in Membrane after the Addition of the Nanoparticle
Zeolites	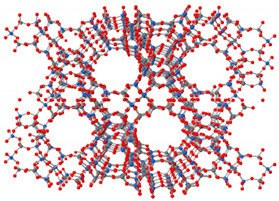	Hydrophilicity, filtration, tunable chemistry molecular sieve, and high permeability
Magnetite	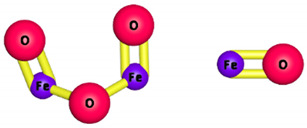	Superparamagnetic and tunable chemistry
Silver	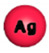	Anti-biofouling and antimicrobial
TiO_2_	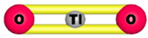	Chemical stability, reactivity, photocatalysis, and hydrophilicity
Carbon nanotubes (CNTs)	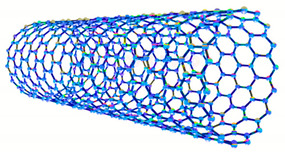	Chemical stability, tunable chemistry, antimicrobial, high mechanical strength, and anti-biofouling

**Table 2:** A summary of key elements of TiO_2_ nanoparticle-based nanocomposite membranes.

Membrane Application	Modification Technique	Membrane Modification Enhancement	Reference
Study of Escherichia coli	Dipped coating	Anti-bio-fouling property was improved.	[43]
membrane bioreactor system	Dipped coating	Higher anti-fouling properties	[44]
activated sludge filtration	Dipped coating and Phase inversion	Increase in composite membrane porosity, and a higher anti-fouling properties.	[45]
Treatment of emulsified oil wastewater	Phase inversion method	Higher water permeability, hydrophilicity, mechanical strength and anti- fouling ability	[46]
Enhancement of PES/PI nanofiltration membranes	Dipped coating under UV	High flux recovery	[47]
Study of the performance of PVDF membrane	Phase inversion method	Enhanced antifouling properties of PVDF (polyvinylidene fluoride) membrane	[48]
The synthesized membrane can be used as an advanced filtration system	Sol-gel method/Deep coating method	Higher mechanical strength and structural stability.	[49]
Alkaline fuel cells (AFC)	Phase inversion method	Greater thermal properties, thermal resistance and enhanced water take.	[50]
Study of the morphology and properties of poly(phthalazine ether sulfone ketone) (PPESK)	Phase inversion method	Enhanced antifouling properties, increase in tensile mechanical properties, higher membrane hydrophilicity and wettability.	[51]
Removal of harsh organic solvents	Phase inversion method	Higher antifouling property, thermal stability, and flux recovery.	[52]
Study of poly (vinylidene fluoride) (PVDF)/sulfonated polyethersulfone (SPES) blend membrane	Dipped coating	Higher long-term flux stability and antifouling property.	[53]
Study of Polyethersulfone ultrafiltration membranes	Surface deposition in presence and absence of UV	Reduction in membrane fouling.	[54]
Study of PES/TiO_2_ composite membranes	Phase inversion method	Improvement in thermal stability, hydrophilicity, mechanical strength and anti-fouling property.	[55]
Study of the polysulfonamide/nano titanium dioxide (PSA/nano-TiO_2_) composite	Phase inversion using a spinning technique	Better thermal stability and greater ultraviolet resistance	[56]
Membrane can be used in guided bone regeneration (GBR)	Casting method	Greater mechanical strength, and higher antimicrobial activity	[57]
Study the photo-bactericidal effect on *Escherichia coli (E. coli)*	Phase inversion method	Better antibacterial property, higher hydrophilicity, greater flux recovery and enhanced antifouling property.	[58]
Study of titania nanocomposite polyethersulfone ultrafiltration membranes	The sol-gel surface coating method	Higher stability, durability, hydrophilicity, and antifouling property	[59]
Degradation of dyes	Phase inversion using electro-spinning	Greater photocatalytic activity	[60]
Improving fouling resistance	Phase inversion method	Greater permeability, higher antifouling property and improved hydrophilicity	[61]
Study of sulfonated-polyethersulfone (SPES)/nano-TiO_2_ composite UF membrane	Casting method	Greater antifouling property, improved photocatalytic activity and binding strength	[62]
Study of polyamide thin film nanocomposite (TFN) nanofiltration membrane	Surface coating	Higher salt rejection, permeability, thermal stability, and selectivity.	[63]
Study of photocatalytic polyvinyl alcohol (PVA)/TiO_2_ composite polymer membrane	Phase inversion method using electro-spinning	Higher photocatalytic activity, and enhanced tensile strength	[64]
Study of PVDF membrane	Dipped coating	Higher antifouling property	[65]
Study of microporous PES membrane	Phase inversion method	Greater thermal stability, and permeation. In addition, the pore size of the membrane surface layer and the breaking strength was increased.	[66]

**Table 3:** A summary of key elements of SiO_2_ nanoparticle-based nanocomposite membranes.

Membrane Application	Modification Technique	Membrane Modification Enhancement	Reference
Polyethersulfone-mesoporous silica nanocomposite ultrafiltration membranes	Phase inversion casting method	Greater hydrophilicity, thermal stability, porosity, water uptake and antifouling properties.	[67]
Polysulfone/silica nanoparticle mixed-matrix membranes used for gas separation	Phase inversion method	Enhanced gas permeability of the PS (polysulfone) membrane	[68]
Ce-doped nonstoichiometric nanosilica/polysulfone composite membranes used in wastewater treatment	Phase inversion method	Greater tensile strength, antifouling property, and hydrophilicity.	[69]
Poly(vinylidene fluoride) composite membranes applied in the electro-driven separation processes	Phase inversion method	Higher conductivity, selectivity, and physical stability.	[70]
Organic/inorganic composite membranes	Solution casting method	Higher chemically stability and tensile strength. In addition, the membrane proton conductivity was also improved	[71]
PDMS nanocomposite membranes used for gas separation	Casting method	Greater permeability	[72]
PSf/SiO_2_ nanocomposite membrane applied in oil-in-water emulsion separation	Phase inversion method	Higher permeability and antifouling property.	[73]
Silica nanocomposite membranes	Phase inversion method	Increase in water diffusivity and fractional free-volume.	[74]
Nanocomposite membranes for gas separation	Phase inversion method	Higher diffusivity, gas permeability, solubility, and selectivity.	[75]
Nano silica/Nafion composite membrane applied in proton exchange membrane fuel cells	Phase inversion method	Higher proton conductivity.	[76]
Polymer Nanocomposite Electrolyte Membrane used for High Performance Lithium/Sulfur Batteries	Casting method	Higher electrochemical stability, and ionic conductivity.	[77]
PBI and PBI/ZIF-8 nanocomposite membranes	Phase inversion method	Improved solubility, degree of swelling, and selectivity	[78]
PVA/nano silica composite membranes	Phase inversion method	Higher hydrophilicity and flux.	[79]

**Table 4:** A summary of key elements of several nanoparticle-based nanocomposite membranes.

Nanoparticle	Membrane Application	Modification Technique	Membrane Modification Enhancement	Reference
Zeolite	Polymer-Zeolite Nanocomposites as Mixed-Matrix Membranes used for Gas Separation	Casting method	Greater permeability for CH_4_, N_2_, and CO_2_.	[80]
ZIF-8	Polybenzimidazole (PBI) and PBI/zeolitic imidazolate framework (ZIF-8) nanocomposite membranes	Phase inversion method	Increase in permeability, sorption diffusion coefficient, pervaporation, and swelling characteristics.	[78]
ZnO	Chitosan/ZnO nanoparticle composite membranes	Phase inversion	Higher antibacterial property and mechanical stability.	[81]
Al_2_O_3_	Al_2_O_3_/PES membrane applied in wastewater filtration	Phase inversion	The composite membrane had a decline in the fouling effect and a decrease in flux.	[82]
SiO_2_-Al_2_O_3_	Nanocomposite SiO_2_-Al_2_O_3_ membrane	Surface coating	Higher structural stability and hydrogen selectivity.	[83]
Zirconia	Poly(arylene ether sulfone)/Nano-ZrO_2_ Composite Anion Exchange Membranes applied in Alkaline Fuel Cells	Phase inversion	Improved water uptake, hydroxide ion conductivity, dimension stability, mechanical properties, thermal stability and chemical stability.	[84]
ZrO_2_, Al_2_O_3_, and TiO_2_	Nano-structured ceramic–metallic composite microporous membranes for gas separation application	Spray assisted surface coating	Enhanced thermal and chemical stability.	[85]
Al_2_O_3_	Hybrid Composite Membranes used for Lithium-Ion Batteries	Dipped Coating	Greater thermal stability and enhanced wettability.	[86]
Fe/Pd	Microfiltration Membrane	Ion-exchange pore diffusion technique	Higher reactivity.	[87]
ZnO	PVDF microfiltration membranes used for water treatment	Phase inversion	The composite membrane had greater water flux, breaking strength, and pore size distribution.	[88]

**Table 5:** Summary of nanomaterials used in membranes for water/wastewater treatment.

Nanomaterial	Application in Water/Waste Treatment	Process Applied	Enhancement in Membrane after the Incorporation of the Nanomaterial
CNTs, zeolites, metal-oxides and chitosan	Pollutant removal	Adsorption	High surface area, high accessible adsorption sites, fine-tuning of compound to pollutant, easy to reuse
nZVI, Au, and TiO_2_	Pollutant degradation	Photocatalysis or chemical reduction	Catalytic reduction and photocatalysis not seen in bulk materials, unique quantum effects
Chitosan, Ag, TiO_2_ and MgO, and CNTs	Removal of contaminants from drinking water or wastewater	Disinfection	Cell membrane damage, metal chelation in cells, reactive oxygen species (ROS) production, chemical stability

**Table 6:** Application of nanoparticles in the removal of contaminants from water.

Nanoparticle	Contaminants	Removal Capacities	Rejection (%)	Process Used	pH	Contact Time	Reference
Aluminium substituted goethite (Al-FeOOH)	Ni	94.52 mg·g^−1^	-	-	5	6 h	[103]
SiO_2_	Oil/water emulsion	-	99%	Microfiltration	-	-	[104]
ZnO and montmorillonite	Cu(II)	-	-	-	4	90 min	[105]
AgNps	*E*. *coli*, *B*. *subtilis*		94%	Microfiltration	-	-	[106]
Iron nanoparticles modified micro fibrillated cellulose	As(V)	2.460 mmol·g^−1^	-	-	2	75 min	[103]
Hematite	As(III) and As(V)	2899 ± 71.09 μg·g^−1^ and 4122 ± 62.79 μg·g^−1^	-	-	6–8	8 h	[107]
Nanoscale zero valent iron (NZVI)	Cr(VI)	100%	-	-	2	10–30 min	[108]
TiO_2_@g-C_3_N_4_	tetracycline	-	97%	Photocatalysis	-	-	[109]
Magnetite Fe_3_O_4_/Chitosan nanoparticles (Fe_3_O_4_/CSNPs)	Pb(II)	79.29 mg·g^−1^	-	-	6	12 h	[110]
MWCNTS, Graphene, TiO_2_	Cadmium	-	100%	Adsorption	-	-	[109]
MgO	Pb(II)	2614 mg·g^−1^	-	-	-	180 min	[111]
Zerovalent iron and reduced graphene oxide	Cd(II)	425.72 mg·g^−1^	-	-	5	50 min	[112]
CNTS	TOC	-	30.5%	Microfiltration	-	-	[113]
Ascorbic acid-stabilized zero valent iron Nps	Cd(II)	79.58%	-	-	7	60 min	[114]
Copper oxide	Cr(VI)	15.62 mg·g^−1^	-	-	3	180 min	[115]
Ag Nps	AZG dye		85%	Photocatalysis			[116]
Graphene oxide-Cobalt oxide	Cr(VI)	208.8 mg·g^−1^	-	-	3	12 h	[117]
γ-Al_2_O_3_ NPs	Cd(II)	17.22 mg·g^−1^	-	-	5	30 min	[118]
Manganese ferrite and cobalt	As(III)	24.17 and 24.81 mg·g^−1^	-	-	2	4 h	[119]
ZnO Nps	Oil, *E. coli*	-	–	Microfiltration, Antimicrobial	-	-	[120]
Sulfonated magnetic NPs	Pb(II)	108.93 mg·g^−1^	-	-	7	24 h	[121]
γ-alumina NPs and MWCNTs	Ni	99.41% and 87.65%	-	-	10	30 min	[122]
Titanate nanotubes	Th(I) and Th(III)	709.2 mg·g^−1^	-	-	-	10 min	[123]
OMWCNTs	*Indigo*		98%	Microfiltration	-	-	[124]
Modified henna with Fe_3_O_4_	Cu(II)	99.11%	-	-	4	85 min	[125]
SiO_2_	Oil/water	-	98%	Microfiltration	-	-	[126]
γ-alumina	Cu(II)	31.3 mg·g^−1^	-	-	5	4 h	[127]
Fe_3_O_4_	Ni	209.205 to 362.318 mg·g^−1^	-	-	8	35 min	[107]
GO	Oil/water; Methylene Blue dye	-	99%, 95.38%, 92.45%	Microfiltration, Adsorption	-	-	[128]
Nanoscale zero valent iron (nZVI)	Pb(II), Cd(II), Cu(II), Ni(II)	-	-	-	2–7	30 min (Pb), 20 min (Cd, Cu, Ni)	[129]

**Table 7:** Application of modified nanocellulose membranes in the removal of contaminants for water treatment.

Modified Nanocellulose	Method Used	Application	Removal Efficiency	Reference
Amino-modified CNF	Infusion	Microfiltration of virus, bacteria, and metal ions adsorption	MS2: LRV 4; *E. coli*: LRV 6; Metal ions: -	[153]
TEMPO-oxidized CNC	Membrane coating	Metal ions adsorption	–	[154]
BTCA-functionalized CNC	Spray coating	Metal ions adsorption	58.05%	[155]
TEMPO-modified and Unmodified CNF	Membrane deposition	Oil-water separation	>99%	[156]
Meldrum’s acid-modified CNF	Impregnation	Dye adsorption and Microfiltration of Fe_2_O_3_ nanoparticles	>99% dye and nanoparticles	[157]
TiO_2_-modified CNC	In-situ growth	Oil-water separation	>99.5%	[158]
AgNP- and PtNP-grafted CNC	phase separation	wastewater treatment	92−94%	[159]
(tridecafluoro-1,1,2,2-tetrahydrooctyl)-trichlorosilane-modified BNC	Supercritical-drying	Desalination using DCMD	>99.8%	[160]
Thiol-modified CNF	Infusion	Metal ions adsorption	>93%	[161]
Alkoxysilanes-modified BNC	Conventional drying	Water-oil separation	>99%	[162]
Ag-modified CNF	Immobilizations	Dye degradation	98%	[163]
(3-aminopropyl) triethoxysilane-modified BNC	Freeze-drying	Metal ion adsorption	5–100%	[164]
Fe_3_O_4_ modified CNF	In-situ synthesis	Dye degradation	94.9%	[165]

**Table 8:** Application of various nanofibrous membranes in the removal of heavy metals from water for water treatment.

Nano Fibrous Membrane	Heavy Metal Ion	Adsorption Capacity (Mg/G)	Reference
Chitosan	As(V)	11.2	[166]
Multiwalled carbon nanotube-Polyethyleneimine/Polyacrylonitrile	Pb(II), Cu(II)	232.7, 112.5	[167]
Polyindole	Cd(II)	140.36	[168]
Polyvinyl alcohol/Silica	Cu(II)	489.12	[169]
Silk fibroin/Cellulose acetate	Cu(II)	22.8	[170]
Polyvinyl alcohol/Titanium dioxide/Zinc oxide	Th(IV)	333.3	[171]
Chitosan	As(V)	30.8	[172]
Polyacrylonitrile/Titanium dioxide	Pb(II), Cd(II)	193, 91	[173]
Chitosan/Cellulose acetate	Cd(II)	110.48	[174]
Polyvinylpyrrolidone/Silica/3-Aminopropyltriethoxysilane	Cd(II), Pb(II), Ni(II)	157.4, 158.3, 63.0	[175]
Chitosan	Cr(VI)	20.5	[176]
Polyamide 6/Fe_3_O_4_/Oxidized multiwalled carbon nanotubes	Pb(II)	49.3	[177]
Wool keratose/Silk fibroin	Cu(II)	2.88	[178]
Polyvinylpyrrolidone/Silica	Cr(III)	97	[179]
Chitosan/poly(L–lactic acid)	Cu(II)	111.66 ± 3.22	[180]
Polyvinyl alcohol/Titanium dioxide	Th(IV)	238.1	[181]
polyethersulfone-poly (dimethyl amino) ethyl methacrylate	Cu(II)	161.3	[182]
Chitosan/Polyvinyl alcohol	Cu(II)	90.3	[183]
Polyacrylonitrile/Fe_2_O_3_/Sodium dodecyl sulfate	Cu(II), Pb(II), Cd(II)	11.8, 30, 7.5	[184]
Chitosan/Poly(ethylene oxide)/Permutit	Cr(VI)	208	[185]
Polyacrylonitrile/γ-AlOOH	Pb(II), Cu(II), Cd(II)	180.83, 48.68, 114.94	[186]
Polyethyleneimine/Polyvinyl alcohol	Cr(VI)	150	[187]
Polyacrylic acid/Polyvinyl alcohol/Zero-valent iron	Cu(II)	107.8	[188]
Chitosan/Graphene oxide	Cu(II), Pb(II), Cr(VI)	461.3, 423.8, 310.4	[189]
Polyethyleneimine/Polydopamine	Cu(II)	33.59	[190]
Polyetherimide-Fe_3_O_4_/Polyacrylonitrile	Cr(VI)	684.93	[191]
Chitosan/Sodium polyacrylate	Cr(VI)	78.92	[192]
Polyvinyl alcohol/Chitosan/ZnO	Cd(II), Ni(II)	138.77, 50.21	[193]
Polyindole	Cu(II)	121.95	[194]
Poly(vinylidene fluoride)/Polydopamine	Cu(II)	26.7	[195]
Wool keratin/Nylon 6	Cu(II)	103.5	[196]
Polyacrylonitrile/Cellulose acetate/ZIF-67	Cu(II), Cr(VI)	18.9, 14.5	[197]
Chitosan/Poly(ethylene oxide)	Ni(II)	227.27	[198]
Polyvinyl alcohol/NaX zeolite	Ni(II), Cd(II)	342.8, 838.7	[199]
Polyacrylic acid/Polyvinyl alcohol	Pb(II)	288	[200]
Polyvinyl alcohol/Sb-TBC Polyvinyl alcohol/Sr-TBC Polyvinyl alcohol/La-TBC	Pb(II)	91 124 194	[201]
Polyacrylonitrile/Polypyrrole	Cr(VI)	74.91	[202]
Cellulose acetate/Polymethacrylic acid	Pb(II)	146.21	[203]
Polyacrylic acid/Sodium alginate	Cu(II)	591.7	[204]
Polystyrene/Titanium dioxide	Cu(II)	522	[205]
Chitosan/Titanium dioxide	Cu(II), Pb(II)	710.3, 579.1	[206]
Polyacrylonitrile/Zinc oxide	Pb(II), Cd(II)	322, 166	[207]
Polyacrylonitrile@γ-AlOOH	Cr(VI)	5	[208]
Ethyl cellulose/Al_2_O_3_	Pb(II)	134.5	[209]
Silica@Polyvinylidene fluoride-hexafluoropropylene	Cu(II)	21.9	[210]
Polyacrylonitrile/Chitosan	Cr(III)	116.5	[211]
MgAl-EDTH-LDH@Polyacrylonitrile	Cu(II)	120.77	[212]
Polyvinyl alcohol/Silica	Mn(II), Ni(II)	234.7, 229.9	[213]
Polyvinylpyrrolidone/Silica	Hg(II)	852	[214]
Chitosan/Poly (ethylene oxide)/Activated carbon	Cr(VI), Fe(III), Cu(II), Zn(II), Pb(II)	261.1, 217.4, 195.3, 186.2, 176.9	[215]
Poly (ethylene oxide)/Graphene oxide	Cu(II), Cd(II)	44.7, 59.1	[216]
Cellulose/Graphene oxide	Hg(II)	13.73	[217]
Polyacrylonitrile/F300 Polyacrylonitrile/MOF808 Poly(vinylidene fluoride)/MOF808	Hg(II), Pb(II)	53.09, 30.19 50.88, 23.98 42.60, 17.19	[218]
Polyacrylonitrile/MOF-808	Cd(II), Zn(II)	225.05, 287.06	[219]
Chitosan/Polyvinyl alcohol/Zeolite	Cr(VI)	450	[220]
Chitosan/Fe	As(III)	36.1	[221]
Chitosan/Fe_3_O_4_/Oxidized multiwalled carbon nanotubes	Cr(VI)	358	[222]

**Table 9:** Application of graphene and its derivatives in the removal of heavy metals and dyes from water for water treatment.

Adsorbent	Pollutant	Adsorption Capacity (mg·g^−1^)	Kinetic Model	Reference
Reduced graphene oxide (rGO) decorated with molybdenum disulfide (MoS_2_)	Cr(III)	242	-	[235]
	Co(II)	112		
	Ni(II)	145		
	Cu(II)	417		
	Zn(II)	550		
	Pb(II)	498		
Chitosan reinforced graphene oxide-hydroxyapatite (CS@GO-Hap)	Congo Red (CR)	43.06	pseudo-second-order	[236]
	Acid Red 1 (AR1)	41.32		
	Reactive Red 2 (RR2)	40.03		
β-CD/PAA/GO nanocomposites	methylene blue (MB) safranine T (ST)	247.99 175.49	Langmuir	[237]
MnO_2_ nanotubes@reduced graphene oxide hydrogel (MNGH)	Pb^2+^	356.37	-	[238]
	Cd^2+^	177.4		
	Ag^+^	138.2		
	Cu^2+^	121.5		
	Zn^2+^	83.9		
Graphene oxide embedded calcium alginate (GOCA)	Pb(II)	602	Pseudo-second-order	[239]
	Hg(II)	374		
	Cd(II)	181		
Silica-decorated graphene oxide (SGO)	Cadmium(II)	43.45	pseudo-second-order	[240]
Thiosemicarbazide functionalized graphene oxide (GO-TSC-GO)	methylene blue (MB)	596.642	pseudo-second-order	[241]
Fe_3_O_4_/SiO_2_-GO	Cd(II) Pb(II)	128.2 385.1	-	[242]
Poly(m-phenylenediamine)/reduced graphene oxide/nickle ferrite nanocomposite	Cr(VI)	502.5	pseudo-second-order	[243]
Graphene oxide–silica composite	Congo red (CR) Cadmium(II)	43.45 333.33	pseudo-second-order	[240]
Graphene oxide-activated carbon (GO-AC) composite	methylene blue (MB) crystal violet (CV)	147 70	pseudo-second-order	[244]
Graphene oxide (GO)	Pb^2+^	75.41	pseudo-second-order	[245]
	Ni^2+^	29.04		
	Cd^2+^	31.35		
Reduced graphene oxide (rGO)	malachite green (MG)	476.2	pseudo-second-order	[246]
GO@SiO_2_-MSp@SiO_2_NH_2_	Pb(II)	323.5	pseudo-second-order	[247]
Reduced graphene oxide/Lanthanum Alluminate nanocomposites (RGO-LaAlO_3_)	Methyl orange (MO)	702.2	Pseudo-second-order	[248]
Sulfonated graphene oxide (SGO)	Pb^2+^	415	Pseudo-second-order	[249]
MnFe_2_O_4_/rGO magnetic nanoparticles (MRGO)	methylene blue (MB)	105	Pseudo-second order	[250]
Graphene oxide functionalized chitosan-magnetite nanocomposite	Cu(II) Cr(VI)	111.11 142.85	Pseudo-second-order	[251]
Fe_3_O_4_/graphene nanocomposite	Cr(VI)	280.6	Pseudo-second-order	[252]
magnetic CoFe_2_O_4_/graphene oxide (GO)	methylene blue (MB) rhodamine B (RhB)	355.9 284.9	Pseudo-second-order	[253]
Graphene oxide (GO)	Pb(II)	555	Pseudo-second-order	[254]
Bimetal oxide decorated graphene oxide (Gd_2_O_3_/Bi_2_O_3_@GO) nanocomposite	Methyl orange (MO)	544	Pseudo-second-order	[255]
Thiosemicarbazide-grafted graphene oxide (GO-TSC)	Hg(II)	231	-	[256]
3D graphene nanoedges	methyl orange (MO)	27.932	-	[257]
Porous silica–graphene oxide nanocomposite(GO-SiO_2_)	Pb(II) As(III)	527 30	Pseudo-second-order	[258]
Magnetic CoF/GO	MB	157	Pseudo-second-order	[259]
	MV	122		
GN-MnO_2_	Co(II) Cr(III)	403.4 491.98	Second-order-pseudo	[260]
Graphene oxide	Congo Red (CR)	120.20	second order	[261]
Bifunctionalized graphene oxide/MnFe_2_O_4_ magnetic nanoparticles (PEHA-Phos-GO/MnFe_2_O_4_)	Pb(II)	366.4	Pseudo-second-order	[262]

## Data Availability

Not applicable.
